# Shared principles, local practice: an international survey of child death review systems

**DOI:** 10.1136/bmjpo-2026-005019

**Published:** 2026-09-08

**Authors:** Merve Tosyali, Jeanine Young, Joanna Jane Garstang

**Affiliations:** 1 Ege University Faculty of Medicine, Izmir, Mağaza Seçiniz, Turkey; 2 School of Nursing, Midwifery and Paramedicine, University of the Sunshine Coast, Maroochydore, Queensland, Australia; 3 School of Health Sciences, University of Birmingham, Birmingham, UK; 4 Children and Families Division, Birmingham Community Healthcare NHS Foundation Trust, Birmingham, UK

**Keywords:** Child Health, Health Policy, Mortality

## Abstract

**Objective:**

Child death review (CDR) is the multiprofessional review of child deaths aiming to identify causes, risk factors and learning, with the aim of improving services and preventing future deaths.

The objective of this study was to determine the range of current CDR practice internationally.

**Design:**

This study used a descriptive comparative survey design to determine similarities and differences between CDR practices in different countries. A secure online questionnaire was developed by the CDR working group of the International Society for the Study and Prevention of Perinatal and Infant Death and distributed via international professional networks.

**Setting:**

Professionals working in CDR in any country globally, contacted via email.

**Participants:**

27 professionals from 19 different countries participated in the study. To ensure data consistency, duplicate responses from the same region within countries were combined. A total of 20 responses from regions within 13 different countries were included.

**Outcomes:**

Key measures included legal status of CDR systems, family involvement in CDR, legal protection for professionals, multidisciplinary team structure and CDR inclusion criteria.

**Results:**

CDR was identified in 13 countries; 5 describing national CDR programmes and 8 with regional programmes. The aim of all CDR programmes was to prevent future deaths. Laws mandating CDR existed in 15 regions or countries; areas without legislative support reported challenges in conducting CDR. Parental involvement in CDR varied considerably between regions: six required parental consent for reviews, while nine did not inform parents about reviews. In 10 regions, all child deaths regardless of cause were included in CDR, while other programmes reviewed only selected deaths. In seven regions, professionals were provided with legal protection from malpractice claims to enable participation in the CDR.

**Conclusions:**

Common principles underpin effective CDR, providing a foundation for locally adapted approaches to improve systems and prevent future deaths.

**WHAT IS ALREADY KNOWN ON THIS TOPIC**

- Child death review (CDR) is the multiprofessional process for learning from deaths, aiming to prevent future deaths.
- Many countries have CDR programmes but there is no international consensus on how reviews should be conducted or which deaths should be reviewed.

**WHAT THIS STUDY ADDS**

- This study details CDR practices in several countries, showing the variation in inclusion criteria, involvement of parents, legal basis for reviews and malpractice protection for professionals.

**HOW THIS STUDY MIGHT AFFECT RESEARCH, POLICY OR PRACTICE**

- Wide variation in CDR practices highlights a need for research establishing which structural elements (eg, inclusion criteria, shared principles, legislative mandate, professional discipline involvement) most effectively support prevention outcomes to inform harmonised but locally adaptable policy.

## Introduction

 Child death review (CDR) is a multidisciplinary process of detailed investigation of child deaths, grounded in the recognition that every child’s death is a tragedy and that each death can be treated as a sentinel event from which important lessons can be learnt. Learning from child deaths is critical because, although rare, many are preventable and may reveal unrecognised systemic gaps, service failures, or environmental hazards. Systematic review of these deaths enables identification of patterns and contributing factors to inform improvements in policy, practice and product safety, with the aim of strengthening safeguarding systems and reducing likelihood of future deaths.^1^

The primary purpose of CDR is to identify risks and modifiable factors that contributed to death in order to develop effective prevention strategies, although in some jurisdictions CDR is also used to determine cause of death.^1–3^ The process is underpinned by respect for the rights of the children and families and has historically evolved from inquiries into child abuse deaths. In the UK, this stemmed from the 1974 UK review of the death of Maria Colwell, which contributed to the development of modern multiagency child protection systems. Alongside the focus on child abuse deaths, the UK conducted confidential national enquiries into sudden unexpected death in infancy^4^ and child deaths,^5^ leading to the establishment of Child Death Overview Panels in 2008, which review all child deaths. Similarly, in the USA, Child Fatality Review Teams were first established in 1978 in Los Angeles to review child abuse deaths,^6^ with over 1350 multidisciplinary teams now reviewing a wide variety of deaths.^7^ The WHO guide for audit and review of paediatric mortality promotes reviewing deaths as a mechanism to improve the quality of paediatric health systems, particularly in low-income and middle-income countries.^8^ A survey conducted by the International Society for the Prevention of Child Abuse and Neglect (ISPCAN) in 2019–2020 found that 27 of 62 participating countries had some form of child fatality review, although there were no details on the systems or methods of review.^9^ However, this survey was limited to ISPCAN member countries, and there are large parts of the world under-represented.

Although child deaths have declined significantly over the last century, serious disparities remain between countries and within countries.^10^ International child mortality data are generally derived from death certificates, which provide minimal context about context or contributing factors,^11^ limiting learning and opportunities for prevention. Data from CDR in many countries have been used to inform campaigns to reduce the risk of sudden unexpected infant death (SUID), child protection practices and suicide. In Australia, CDR findings on sleep-related SUID risks, including hazards associated with infant sleep products have informed both regulatory and prevention responses, contributing to national mandatory safety and information standards for infant sleep products^12^; and supporting Queensland’s statewide implementation of the Pēpi-Pod Program to reduce risk, particularly among smoke-exposed infants.^13^ High quality CDR should be a public health priority and has recently been endorsed as such by the American Academy of Pediatrics.^7^ The English National Child Mortality Database has conducted many different analyses of child deaths, including those from suicide,^14^ asthma^15^ and life-limiting conditions.^16^ The US National Fatality Review—Case Reporting System has supported several analyses, for example, deaths from suicide,^17^ drowning^18^ and opioid poisoning.^19^ These show the potential value of detailed CDR and regional or national data collection systems.

The CDR process is multidisciplinary in nature and encompasses a wide range of professionals, including paediatricians, family health specialists (including midwives and nurses), pathologists, police, child welfare officers, emergency response teams and education and public health professionals.^1–3^ There is increasing interest in CDR among child health professionals, given the potential benefits in improving prevention strategies. Both ISPCAN and the International Society for the Prevention and Study of Perinatal and Infant Death (ISPID) have CDR working groups, reflecting the origins of CDR in child protection and investigation of SUID. CDR practices vary considerably internationally, with different legal basis, inclusion criteria, professional involvement and outputs. Given this diversity of practice, the primary aim of this study is to compare key contemporary CDR practices to support future development of international best practice guidelines achievable in different countries and regions within countries.

## Methods

This study is a descriptive survey designed to determine the status, scope and operational processes of CDR practices in different countries. The population of interest included representatives of professional disciplines currently involved in CDR in any country.

A secure online survey, using the JISC platform,^20^ was developed and piloted by the CDR working group of ISPID. Questions included whether or not CDR took place in their country, legal basis of CDR, in-country variation, professional participation in CDR, types of deaths reviewed and parental involvement in CDR. Participants were invited to provide any further comments about CDR in their region. Details of the survey are shown in online supplemental appendix 1.

The survey was distributed by email to professional members of ISPID during June to August 2024, via the ISPCAN CDR working group forum, and at the ISPCAN CDR workshop held at Uppsala, Sweden, on 18 August 2024. Recruitment also included snowball sampling, with the email invitation requesting recipients to forward the survey invitation via regional networks to other professionals involved in CDR. All participants gave informed written consent to participate. We directly emailed international leaders in CDR who were known to us regardless of whether they were ISPID or ISPCAN members to ensure we reached those with firsthand knowledge of their national practices. Once eligibility and consent were recorded, survey responses were de-identified to ensure anonymity during analysis. All personal information was kept confidential in accordance with UK General Data Protection Regulation standards.

Countries could provide more than one response as CDR can vary considerably within countries; however, where multiple responses were received from the same region within the country, these were combined into a single response. Responses indicating that the respondent had no knowledge of CDR taking place at all within their country were excluded from further analysis. We used descriptive statistics to present frequency (n) and percentage (%) values based on categorical variables. Free text comments have been presented to illustrate the range and challenges of CDR practices, but these were not detailed enough for formal thematic analysis.

Patient and public involvement was through the ISPID CDR working group, whose membership includes bereaved parents and support organisations; these members contributed to creating the survey.

## Results

We directly approached CDR professionals from 25 different countries via professional networks. Of respondents, 27 eligible responses were received, representing 19 different countries. We combined multiple responses from the same region within each country, resulting in 20 responses for analysis.

### Countries with CDR programmes

**Figure 1:**
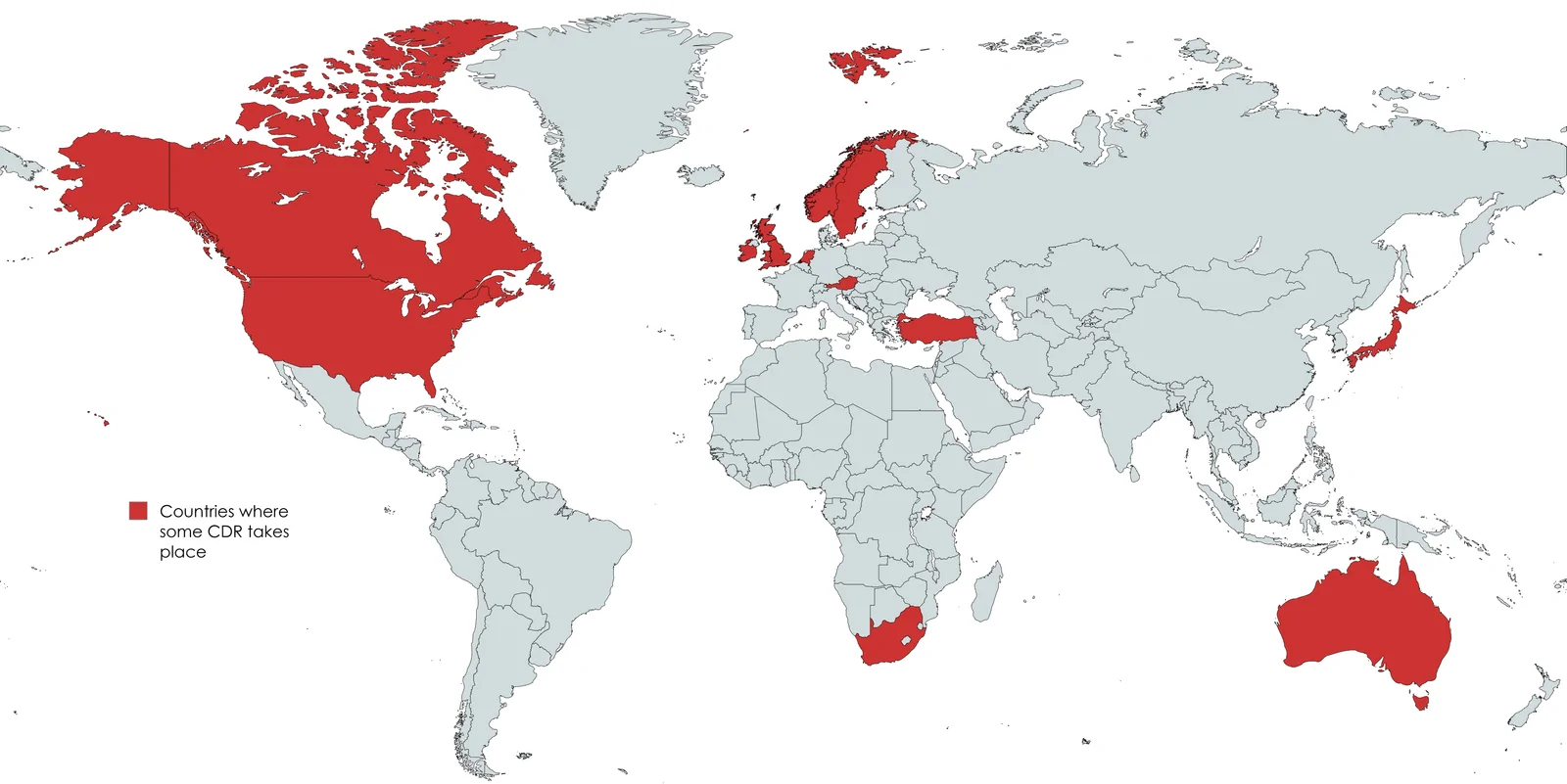
Participant countries which reported child death review (CDR) programmes for some child deaths.

CDR was reported to take place in 13 countries; 6 countries reported no CDR at the time of survey (see figure 1).

Respondents from France, Italy, Northern Ireland, Israel and Chile reported that CDR processes were not yet established but were being considered. Implementation challenges were cited and included legislative gaps, funding restraints, inadequate death investigations and limited information sharing.

CDR implementation varied substantially across jurisdictions. Five countries reported standardised national CDR programmes for some or all child deaths, while eight reported locally or regionally variable practices. Responses from 13 countries (n=20) highlighted differing stages of CDR development, with some jurisdictions having well-established programmes and others in the early phases of implementation.

### Aims of CDR programmes

**Figure 2:**
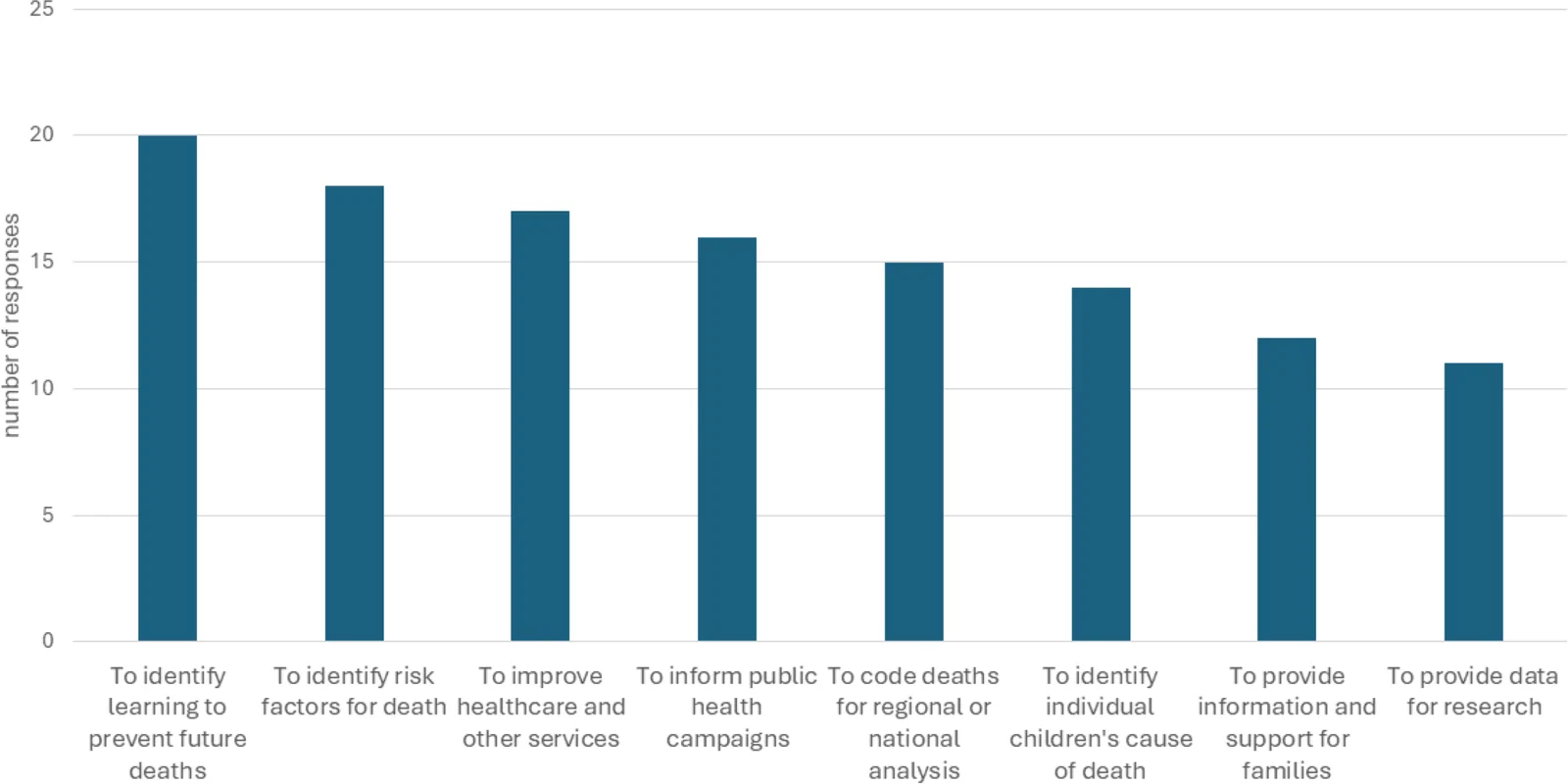
Frequency of different aims for child death review (CDR) programmes.

In all regions, the fundamental objective of CDR was to identify opportunities to prevent future deaths; the different aims of CDR are shown in figure 2.

### Legal basis of CDR

CDR is mandated by regional or national laws in 15 countries or regions; in the remainder, it was described as a voluntary process which could present difficulties in carrying out reviews. The legal basis for CDR in four counties is illustrated in box 1.

Box 1 **Legal basis for child death review (CDR)**

- In England, the 2004 Children Act (amended 2018)^27^ states that “The child death review partners for a local authority area in England must make arrangements for the review of each death of a child normally resident in the area.”
- In the USA, each state has different legislation concerning the structure and function of CDR, and there can be considerable variation within states.
- Australia has a federated approach to CDR, with each state and territory operating under different legislation.^37^ In Queensland, for example, both the Child Death Review Board (Family and Child Commission Act 2014 (Qld))^38^ and Queensland Paediatric Quality Council (Hospital and Health Boards Act 2011 (Qld))^21^ are established through legislation to support protected review and systems improvement processes. In New South Wales (Australia), the role and purpose of CDR are outlined in legislation—Parts 5A and 6 of the Community Services (Complaints, Reviews and Monitoring) Act 1993 (NSW).^39^
- In Japan, respondents felt the lack of CDR legislation is limiting the development of a robust CDR programme due to challenges with information sharing: “We also need a CDR law in order for the law enforcement to be able to share their information in the reviews.”

### Parental involvement in CDR

**Figure 3:**
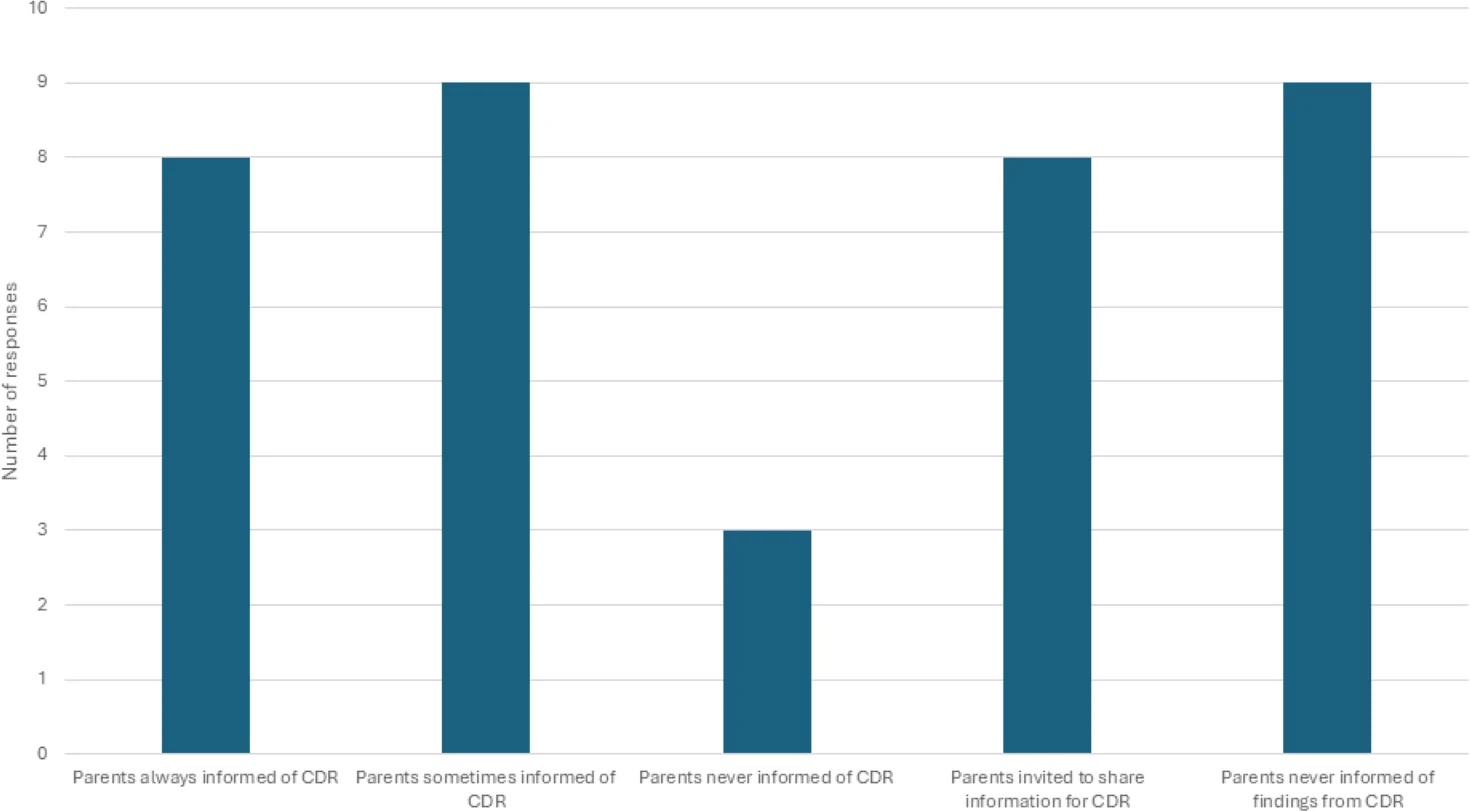
Parental involvement in child death review (CDR) programmes.

Parental involvement in CDR varies considerably, as shown in figure 3.

In England, legislation actively supports parents being informed about CDR, invited to contribute information for reviews and the finding of reviews shared with them.^2^ In the USA, parental involvement varies significantly based on resources, legislation and focus of the CDR team. A parental engagement programme is being developed for CDR teams in the USA to increase parental involvement.

In six regions, parents’ consent is needed prior to any review taking place. In seven regions, professionals have protection from legal claims if malpractice issues are identified during CDR. In the UK, there are no legal protections for professionals contributing to CDR, although the focus is on identifying learning from deaths rather than on accountability. In the USA, legal protections are based on state legislation and vary significantly. The Queensland Paediatric Quality Council, established under the Hospital and Health Boards Act 2011 (Qld),^21^ provides protected clinical review processes where review documents are not legally compellable and confidentiality protections support open multidisciplinary review.^22^

### Types of deaths included in CDR

**Figure 4:**
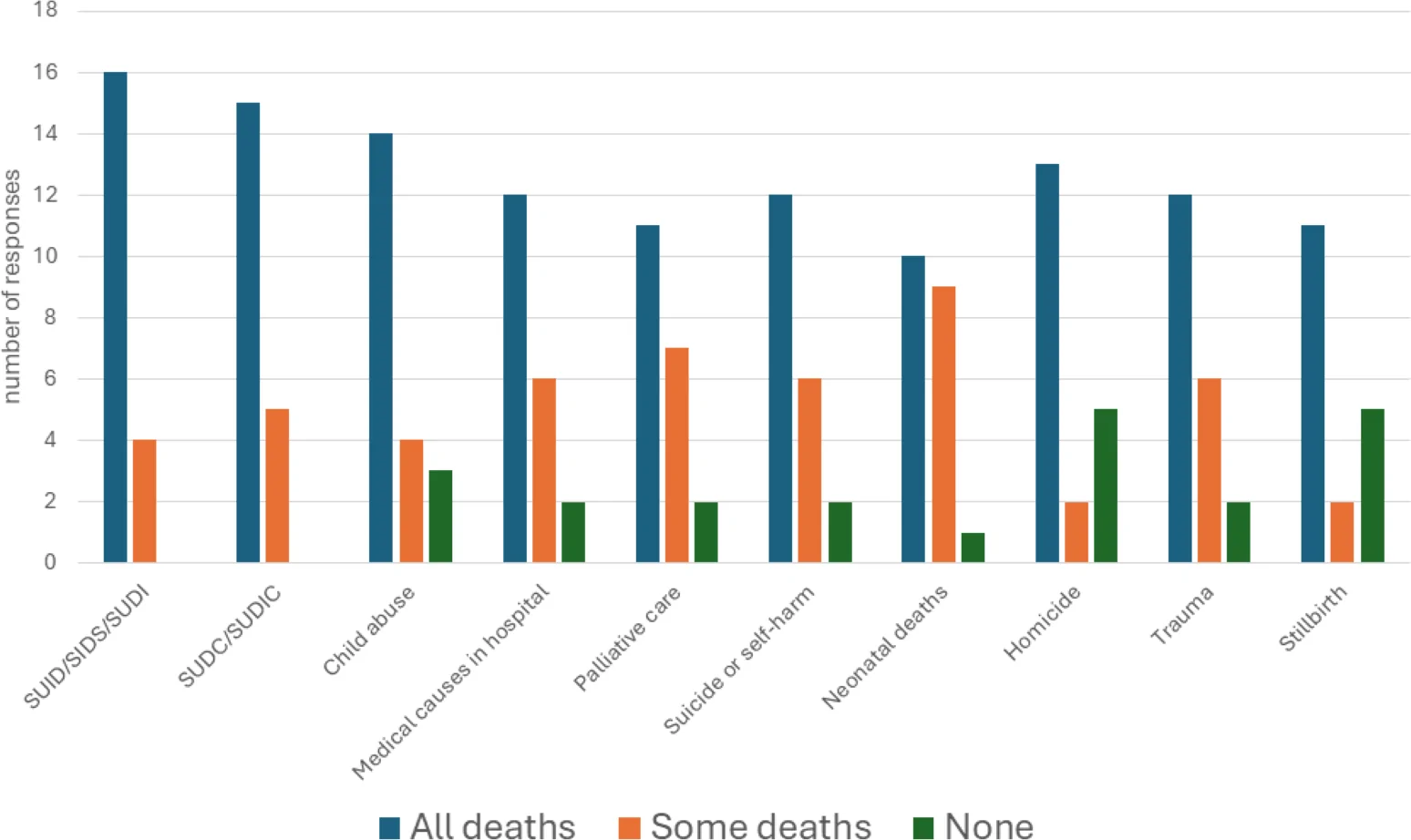
Different types of death included in child death review programmes. SIDS, sudden infant death syndrome; SUDC, sudden unexplained death in childhood; SUDI, sudden unexpected death in infancy; SUDIC, sudden unexpected death in childhood; SUID, sudden unexpected infant death.

The types of deaths included in CDR programmes varied considerably as shown in figure 4.

10 regions reported that all child deaths are reviewed. The most frequently investigated cases included SUIDs, deaths resulting from child abuse or neglect and homicides. In 17 regions, the age of children included in CDR was from birth to 18 years; in three regions, reviews were limited to children under 4 years. In Alberta (Canada) and some US states, CDR extends to include young adults up to the age of 24 who receive social services support.

### Timescales for CDR

13 regions completed reviews within 1 year of death, 6 between 1 and 2 years, with 1 taking more than 2 years to complete reviews.

### Professionals involved in CDR

A range of different multiagency professionals were involved in reviews, with paediatricians, social workers, forensic pathologists and public health personnel most commonly taking part.

### Format of CDR

Standard CDR forms or templates were used in 15 regions to assist reviews; other regions were more flexible in their review processes. The different types of review format used are illustrated in box 2.

Box 2 **Examples of different format for reviews**

- In England, child death review (CDR) follows a standard format supported by an electronic recording system which automatically shares all information with the National Child Mortality Database; this enables thematic analysis and learning nationally.
- In New South Wales (Australia), the focus of reviews varies depending on the underlying cause of death and issues identified, although key demographic information is recorded for all children.
- In Scotland, the format of CDR varies, but there is a national core review data set, which is completed and gathered centrally via an online portal.
- In the USA, the format of reviews varies, but there is a national data system used in all states called the Pediatric National Fatality Review—Case Reporting System that collects comprehensive data and is used to drive analysis and prevention at the regional, state and national levels.

### Outcomes from CDR

The outcomes from CDR vary from determining the cause and risk factors for individual child deaths to thematic learning across several similar cases. Some CDR teams produce annual reports for government. There is national collection of CDR data in 10 countries. The range of outcomes is shown in box 3.

Box 3 **Example outcomes from child death review (CDR)**

- In Queensland (Australia), there is detailed CDR for all infants under 12 months old occurring around 2 years after death following completion of coronial processes. This often leads to a new understanding of the cause of death as more information is available compared with coronial or pathology reports. Annual reports contribute to system recommendations at local, regional and state levels.
- In Alberta (Canada), the CDR team produces an annual report to government on themes identified during reviews, including recommendations to government to address gaps and challenges found. Government departments are required to publicly respond to these recommendations.
- In England, local Child Death Overview Panels produce an annual report detailing key learning and actions taken. The National Child Mortality Database produces national thematic reports with recommendations for government, healthcare services and other agencies.
- In the USA, some state and regional CDR teams produce their own reports. At a national level, data are aggregated and shared in peer-reviewed publications, data infographics and programmatic reports.

## Discussion

This survey reports on CDR practices in 13 countries. Half the participant countries undertaking CDR were reported as having no national system or national data collection, with review practices varying regionally within countries. In all, the overall aim of CDR was to identify learning to prevent future deaths. In most areas, CDR is mandated by regional or national laws. Parental involvement varies significantly; and some regions require parental consent for CDR. 10 regions reported that all child deaths are reviewed. Most used standard CDR forms or templates to support reviews.

One of the key findings of our study is that all participants concurred that the aim of CDR was to identify learning to prevent future deaths, fitting with the principle of viewing every child death as a sentinel event. Nearly all also considered identifying risk factors for deaths as an aim of CDR reflecting professionals’ efforts to go beyond medical classification and develop a deeper understanding of how and why deaths occur. Following changes to the English CDR system that introduced detailed reviews for all children, the proportion of deaths with modifiable factors identified increased from 31% in 2020 to 48% in 2025.^23^ CDR outputs are widely used to develop new policies and inform the public of system level opportunities and recommended strategies for improvement.

Despite a shared purpose, CDR practices remain strikingly diverse, consistent with previous reports.^1 24^ This lack of consistency on which deaths are reviewed, review processes and core data elements limit international collaboration and development of global standards. The impact of this variation is evident in the ISPCAN survey, which reported the proportion of child deaths attributed to different categories of abuse; these estimates depend heavily on review inclusion criteria, with substantially lower proportions in countries reviewing all child deaths than in those reviewing only deaths from abuse or neglect.^9^ Harmonised international CDR datasets using common variables could substantially advance understanding of rare causes of child death, including unexplained infant and child deaths. This is particularly important given international variation in the classification of sudden infant deaths,^25^ where factors such as bedsharing are often interpreted as causal despite limited evidence, hindering surveillance and research.^26^ Standardised datasets could also improve understanding of other rare conditions, such as sudden unexplained death in childhood (SUDC), and clarify the complex relationships between ethnicity, social deprivation and child mortality across developed countries.

In many countries, only certain categories of deaths are reviewed; this potentially significantly limits learning. For example, often deaths from natural causes are excluded; however, recent analysis of CDR data for children dying of asthma in England found significant failures in primary healthcare and safeguarding to inform future system improvements.^15^ Robust CDR systems, such as England, have their basis in law^27^; in systems without a legal basis, data collection processes are often voluntary, which hinders the accurate determination of the true rates of critical cases such as child abuse.^7^

Few countries involve parents in CDR, which also potentially limits learning. The WHO paediatric mortality review guidelines refer to ‘sometimes’ interviewing parents but do not clarify when this may or may not be appropriate.^8^ However, parents may have information about their children’s health, illness, healthcare experiences and family history, which can provide valuable context for reviews, including information not captured as part of immediate police investigations. Arguably, parents should also have the right to know the outcomes of reviews, although half of our respondents reported that parents are never informed about CDR or the conclusions. A recent study by Tiemensma and colleagues^28^ identified considerable variations in coronial and bereavement support for families across Australian states and territories following the sudden and unexpected death of a child. There has been recent work in England to support parental involvement in CDR,^29^ with projects to better engage parents and improved clinical history also underway in Australia and the USA. It is of course vital that bereaved families are treated with great sensitivity and compassion as CDR can be intrusive.^30^

Although all respondents reported multidisciplinary participation in CDR, many had a very medical or forensic focus not including family or public health, social work or education. Similarly, the WHO paediatric mortality review guidelines only relate to review of deaths within paediatric healthcare facilities.^8^ This lack of multidisciplinary CDR suggests potential weakness in the analysis of social determinants of health; this is of concern as child mortality is strongly socially determined.^31^ Only a minority of regions reported legal protection for professionals participating in CDR; this could potentially be an obstacle to effective CDR. Fear of medical malpractice lawsuits may lead to participants avoiding sharing system flaws and errors. The provision of this legal protection in the USA supports a more open structure in their systems, focusing on learning from the system as a whole rather than blaming individuals. The WHO considers providing these legal safeguards as vital for CDR to function as a tool for improving service quality rather than a punitive mechanism.^8^ However, other countries such as England have a robust CDR system without any legal protection for participants.

Despite the relatively small sample, our established international collaborations with CDR leaders enabled us to purposively recruit informants with detailed knowledge of CDR policy and practice within their countries or regions, providing confidence that the data accurately reflect the systems described. The international composition of the author team also brought diverse disciplinary and jurisdictional perspectives to the interpretation of findings.

Nevertheless, this study has important limitations. Despite engaging international CDR networks, responses were not obtained from all countries with established CDR systems. Furthermore, our perspectives are predominantly formed by experience in higher-income settings, with South Africa the only participating low-income or middle-income nation. Consequently, our findings are not representative of the full spectrum of global approaches to learning from child deaths.

Although this study focused on formal multidisciplinary CDR systems, we recognise that many countries employ alternative approaches to mortality review that reflect different health priorities and resource settings. For example, the Solomon Islands uses the WHO paediatric mortality audit guidelines to improve the quality of hospital paediatric care.^32^ Verbal autopsy is widely used across low-income and middle-income countries, particularly in Africa and Asia, to determine causes of death where civil registration and medical certification are limited, although it does not generally support the broader multidisciplinary systems learning related to CDR.^33^ Verbal autopsy can be conducted by means of open or closed questions; open questions may give parents more opportunity to tell their story, which could subsequently inform any CDR.^34^ In Mali and Uganda, a community-based CDR programme adapted the UK confidential enquiry methodology to investigate more than 1000 deaths of children under 5 years of age, placing parents’ accounts at the centre of the review process and generating locally relevant recommendations to improve healthcare and treatment-seeking behaviour.^35^ Similarly, recent child death analyses in Odisha State, India, have identified treatment delays contributing to child mortality, demonstrating the value of mortality review across diverse health systems, although the specific review methodology was not described.^36^ These examples highlight that approaches to reviewing child deaths are necessarily shaped by local epidemiology, resources and health system priorities, and underscore the need for future international work to better incorporate perspectives from low-income and middle-income countries and Indigenous communities. Public health interventions have contributed to a significant decline in global child mortality. Robust multidisciplinary CDR processes could play a complementary role in translating findings at local or regional level into state strategies. The WHO paediatric mortality audit and review guidelines focus on improving paediatric healthcare services. The WHO could consider updating this guidance to reflect multidisciplinary CDR and include the voices of bereaved parents; this could help transform CDR systems and help prevent future child deaths.

In conclusion, this study highlights both the diversity of CDR practices internationally and the common principles that underpin them. Across settings, the shared purpose of CDR is to learn from child deaths and identify opportunities to strengthen systems, improve care and prevent future deaths. How these principles are implemented should be shaped by local epidemiology, health priorities, available resources and cultural context, rather than through a single standardised model. Our findings support the value of multidisciplinary review processes that promote learning from systematic factors in a culture of openness and continuous quality improvement, while recognising that alternative mortality review approaches may be more appropriate in some settings. Future international collaboration, including broader representation from low-income to middle-income countries and First Nations communities, could help define core principles for effective CDR while allowing flexibility in their implementation. Such work could inform future WHO guidance and strengthen the contribution of mortality review to child survival efforts globally.

## Supplementary material

10.1136/bmjpo-2026-005019 online supplemental file 1

## Data Availability

Data are available upon reasonable request.
