# Supplementary material for: Shared principles, local practice: an international survey of child death review systems
Source: BMJ Paediatr Open. 2026 Sep 8;10(1):e005019. doi: 10.1136/bmjpo-2026-005019 (PMC13561003; doi:10.1136/bmjpo-2026-005019)
Supplement: Supplementary file 1 [file bmjpo-2026-005019.reviewer_comments.pdf]

PEER REVIEW HISTORY

BMJ Paediatrics Open publishes all reviews undertaken for accepted manuscripts. Reviewers are asked to complete a checklist review form and are provided with free text boxes to elaborate on their assessment. These free text comments are reproduced below.

ARTICLE DETAILS

**TITLE (PROVISIONAL)**	Shared principles, local practice: an international survey of child death review systems
**AUTHORS**	TOSYALI, MERVE<br>Young, Jeanine<br>Garstang, Joanna

VERSION 1 – REVIEW

**REVIEWER NAME**	Sidebotham, Peter
**REVIEWER AFFILIATION**	
**REVIEWER CONFLICT OF INTEREST**	I have previously collaborated with Dr Garstang in research and policy development in child death review in England. I have previously co-authored papers with Dr Garstang and Dr Young.<br>I have not collaborated with any of the authors in any recent work within the past five years, nor was I involved in this current study.
**AI DISCLOSURE**	No, I have not used AI
**DATE REVIEW RETURNED**	22-Jun-2026

**GENERAL COMMENTS**	This is an interesting but limited report of a survey of child death review practices across 13 countries. The report demonstrates the variation in legislation and practice across these 13 countries and between different regions in some countries. The authors call for global standards and best practice guidelines in light of their findings.<br>Overall, I am not convinced that the conclusions are justified and I do not think this survey adds substantially to the findings of Sharon Vincent's comparative study 13 years ago.<br>The major limitations of the study are first, the limited scope of the study and second, a dominant high-income/Western-centric perspective on the authors' interpretation of their findings.<br>In terms of the scope, the study is limited to reporting on just 13 countries from which practitioners responded to the survey. All but two of these are classified as high income countries, the remaining two being upper-middle income. The survey was distributed through two global professional networks: ISPID and ISPCAN. As such, the reach was limited to those individuals who were participating in either of those networks. No attempt was made to identify or contact child death review teams in those countries that did not respond to the survey or were not included in any of the mailings. I am personally aware of at least half a dozen other countries which have or are establishing child death review programmes. As such, the survey cannot be considered comprehensive or representative.<br>The way the paper is written suggests a Western-dominated mindset, with a focus on the issues affecting child mortality in high-income countries. Limited recognition is given to issues of material resources, nutrition, war, migration or infectious diseases or to the needs of indigenous communities. This is disappointing given the authors' backgrounds as I am sure they are aware of these different

	perspectives and could have reflected more on them in writing the paper. The models of child death review reported are based on those developed in the USA and UK, with an emphasis on child abuse fatality reviews and their extension to other external causes of preventable deaths. There is no recognition of other global efforts to monitor and learn from child deaths, including under-5 mortality and perinatal mortality. There is no acknowledgement of the widespread use of verbal autopsy across Africa and parts of Asia.<br>The paper is well written and easy to follow.<br>Abstract: this is clear and concise and covers all main points. The key messages are appropriate.<br>Introduction: this sets the scope and justification for this survey. As highlighted above, the historical background to CDR as presented is rather narrow; no mention is made of its development in USA, nor of other systems globally. It is important to note that, while CDR stemmed in part from reviews of fatal child abuse, its scope is much broader, and in many countries the two types of review may co-exist; other countries/regions review just child abuse fatalities; while yet others combine child abuse fatalities with deaths from all causes. While this is hinted at, it needs to be made more clear.<br>Methods: these are clearly and concisely reported. The methods are appropriate to the objectives, though limited, as highlighted above.<br>Results: the results are presented clearly. It might be helpful to know the response rate: how many professionals from how many countries were emailed as members of ISPID or included in the ISPCAN CDR working group or as part of the Uppsala workshop?<br>I am not convinced that Box 1 adds anything to the results: why are just Styria (Austria) and Japan reported here? Boxes 2, 3 and 4 give a bit more detail, but it is unclear why just four of the 13 countries are included in each.<br>Discussion: Generally, the discussion summarises the key findings, with some limited reflection on these. The authors make some important points about the value of reviewing all child deaths, having a multi-disciplinary approach, and of involving parents, along with the limitations of not having globally comparable data. There is some helpful consideration of the role of legal protection of professionals.<br>It is a bit misleading to state that the paper reports on CDR practices in 21 countries when for five of these the only data presented are that they are planning to develop CDR and facing difficulties in this.<br>In conclusion, the authors state that ‘this study goes beyond documenting the current state of international CDR practices and shows the need for global standards that can ensure that each child death, while remaining a tragedy, are regarded as an opportunity for learning and prevention.’ I am not convinced that it really does go beyond documenting the current state of international CDR practices, nor that it really achieves that given the limited involvement in the survey.<br>I am not convinced that ‘Our findings indicate that child death reviews should function as a quality improvement tool’. Common sense and the defined purpose of CDR would suggest that, but your survey has not provided any evidence that this is a worthwhile aim or that CDR actually achieves it.<br>The statement that ‘Reducing child mortality rates worldwide requires robust CDR by multidisciplinary teams of professionals, transforming CDR data into a state strategy that directly informs legislative processes’ also does not follow from your findings. Most reduction in child mortality worldwide has occurred prior to the establishment of CDR and through public health interventions that have emerged through different processes. To my knowledge, there

	is no evidence so far that CDR can or does reduce child mortality rates in any region.<br>The statement that 'we now need to update the World Health Organization CDR guidelines' may be true, but as you have not reported on or critiqued the current content of those guidelines, nor identified any specific features that need updating, this statement is not justified.

**REVIEWER NAME**	Palusci, Vincent
**REVIEWER AFFILIATION**	NYU Grossman School of Medicine, Pediatrics & Forensic Medicine
**REVIEWER CONFLICT OF INTEREST**	I am a co-presenter with one of the authors at international conferences.
**AI DISCLOSURE**	No, I have not used AI
**DATE REVIEW RETURNED**	22-Jun-2026

**GENERAL COMMENTS**	This is an important review of practice in child death review using empirical data from an international survey of programs. My major issues are (1) the authors need to expand any justification for why child death review should be more similar across jurisdictions with any variations being addressed (are they not adequately serving their constituents, justify reason for survey?), and (2) formatting for the draft needs to be consistent in line with the journal's policies.<br><br>Minor comments:<br>1. it would be helpful to add data form the International Society on the Prevention of Child Abuse and Neglect regarding countries' participating in CDR. Their publication World Perspectives has data on a good number of countries which can be summarized for the introduction;<br>2. The Figure 1 caption should be rephrased to include countries with no CDR;<br>3. Box 2 should include some information about the US National CDR Center and how it amasses data across these different jurisdictions;<br>4. It would be helpful to reorganize and number the issues: (1) Countries with programs, (2) Different aims for programmes, (3) Parental Involvement, (4)Legal claims/issues, (5) types of deaths reviewed; (6) outcomes of CDR, (7) process/ use of standard forms, etc.<br>5. Expand and add separate section for limitations.<br>6. The last paragraph has some good points that need to be brought out in the abstract and introduction. Include the WHO CDR guidelines in the Introduction.<br>7. Add papers from the US National CDR Center (Collier et al., Pediatrics, 2024).<br>8. Use a figure for point 5 info on results in the appendix.<br>9. Were the results of points 16, 18 in the appendix given in the paper?<br>10. Add total N in the figure 2 caption.

**REVIEWER NAME**	Vincent, Sharon
**REVIEWER AFFILIATION**	Leeds Beckett University, School of Health
**REVIEWER CONFLICT OF INTEREST**	No Conflict of Interest
**AI DISCLOSURE**	No, I have not used AI
**DATE REVIEW RETURNED**	23-Jun-2026

**GENERAL COMMENTS**	This is a clearly written paper that will add to the limited evidence base around international variations in child death review practice. I just have one query. The abstract and results section state that participants came from 19 different countries. However, the first line of the discussion section states that the survey reports on CDR practices in 21 countries so it's unclear where this figure comes from.

VERSION 1 – AUTHOR RESPONSE

	Reviewer Comments (PS)	Author responses	Page No.
1	This is an interesting but limited report of a survey of child death review practices across 13 countries. The report demonstrates the variation in legislation and practice across these 13 countries and between different regions in some countries. The authors call for global standards and best practice guidelines in light of their findings.<br>Overall, I am not convinced that the conclusions are justified and I do not think this survey adds substantially to the findings of Sharon Vincent's comparative study 13 years ago.<br>The major limitations of the study are first, the limited scope of the study and second, a dominant high-income/Western-centric perspective on the authors' interpretation of their findings.	Thankyou for these reflections which have been incorporated into our response. We agree that it is important not to overstate these results however wish to present these findings in the context of contemporary practice from countries engaging in international networks and participating in CDR and process improvement.<br>This section has been revised substantially to acknowledge these insights while highlighting contemporary contributions. This section now reads:<br><br>Nevertheless, this study has important limitations. Despite engaging international CDR networks, responses were not obtained from all countries with established CDR systems. Furthermore, our perspectives are predominantly formed by experience in higher-income settings, with South Africa the only participating low- or middle-income nation. Consequently, our findings are not representative of the full spectrum of global approaches to learning from child deaths.<br><br>Although this study focused on formal multidisciplinary CDR systems, we recognize that many countries employ alternative approaches to mortality review that reflect different health priorities and resource settings. For example, the Solomon Islands uses the World Health Organisation child death audit guidelines to improve the quality of hospital paediatric care.[35] Verbal autopsy is widely used across low- and middle-income countries, particularly in Africa and Asia, to determine causes of death where civil registration and medical certification are limited, although it does not generally support the broader multidisciplinary systems	P9-10

		learning related to CDR.[36] In Mali and Uganda, a community-based child death review programme adapted the UK confidential enquiry methodology to investigate more than 1,000 deaths of children under five years of age, placing parents' accounts at the centre of the review process and generating locally relevant recommendations to improve healthcare and treatment-seeking behaviour. [37] Similarly, recent child death analyses in Odisha State, India, have identified treatment delays contributing to child mortality, demonstrating the value of mortality review across diverse health systems, although the specific review methodology was not described. [38] These examples highlight that approaches to reviewing child deaths are necessarily shaped by local epidemiology, resources and health system priorities, and underscore the need for future international work to better incorporate perspectives from low- and middle-income countries and Indigenous communities.<br><br>We have also changed the title of the paper to focus on the shared principles rather than the geography. The new title is<br><br>Shared principles, local practice: an international survey of child death review systems	
2	In terms of the scope, the study is limited to reporting on just 13 countries from which practitioners responded to the survey. All but two of these are classified as high income countries, the remaining two being upper-middle income. The survey was distributed through two global professional networks: ISPID and ISPCAN. As such, the reach was limited to those individuals who were participating in either of those networks. No attempt was made to identify or contact child death review teams in those countries that did not respond to the survey or were not included in any of the mailings. I am personally aware of at least half a dozen other countries which have or are establishing child death review programmes. As such, the survey	Thankyou. Due to research time and personnel resource limitations we focussed our recruitment strategy on two global professional networks to explore variation in international practices, with an additional snowballing sampling strategy to complement recruitment to local and international leaders in CDR regardless of ISPID or ISPCAN membership. We do however acknowledge these networks include predominantly higher-income countries and have made efforts to ensure these limitations and rationale are more explicit in the methods and discussion sections.<br><br>The survey was distributed by email to professional members of ISPID during June to August 2024, via the ISPCAN CDR working group forum, and at the ISPCAN CDR workshop held at	P4

	cannot be considered comprehensive or representative.	Uppsala, Sweden on 18 August 2024. Recruitment also included snowball sampling with the email invitation requesting recipients to forward the survey invitation via regional networks to other professionals involved in CDR. All participants gave informed written consent to participate. We directly emailed international leaders in CDR who were known to us regardless of whether they were ISPID or ISPCAN members, to ensure we reached those with firsthand knowledge of their national practices	
3	The way the paper is written suggests a Western-dominated mindset, with a focus on the issues affecting child mortality in high-income countries. Limited recognition is given to issues of material resources, nutrition, war, migration or infectious diseases or to the needs of indigenous communities. This is disappointing given the authors' backgrounds as I am sure they are aware of these different perspectives and could have reflected more on them in writing the paper. The models of child death review reported are based on those developed in the USA and UK, with an emphasis on child abuse fatality reviews and their extension to other external causes of preventable deaths. There is no recognition of other global efforts to monitor and learn from child deaths, including under-5 mortality and perinatal mortality. There is no acknowledgement of the widespread use of verbal autopsy across Africa and parts of Asia.	Thankyou for sharing this perspective. Our initial discussion was certainly centred around the experiences of Western countries, given these were the respondents in this survey network. However we believe we have now more explicitly addressed the limited generalisability of these results in higher-income settings and have included a discussion of additional known approaches to investigate and learn from child deaths to represent countries not represented in this data to provide important international context:<br><br>Please see response to point 1 for details of new text	P10
4	The paper is well written and easy to follow.<br><br>Abstract: this is clear and concise and covers all main points. The key messages are appropriate.	Thankyou. No changes made.	
5	Introduction: this sets the scope and justification for this survey. As highlighted above, the historical background to CDR as presented is rather narrow; no mention is made of its development in USA, nor of other systems globally. It is important to note that, while CDR stemmed in part from reviews of fatal child abuse, its scope is much broader, and in many countries the two types of review may co-exist; other countries/regions review just child	This section has been expanded as follows:<br><br>The process is underpinned by respect for the rights of the children and families and has historically evolved from inquiries into child abuse deaths, in the UK this stemmed from the 1974 UK review of the death of Maria Colwell, which contributed to the development of modern multi-agency child protection systems. Alongside of the focus on child abuse deaths, the UK conducted	P2

	abuse fatalities; while yet others combine child abuse fatalities with deaths from all causes. While this is hinted at, it needs to be made more clear.	confidential national enquiries into sudden unexpected death in infancy 4 and child deaths 5 leading to the establishment of Child Death Overview Panels which review all child deaths. Similarly, in the USA, Child Fatality Review Teams were first established in 1978 in Los Angeles to review child abuse deaths, 6 with over 1350 multi-disciplinary teams now reviewing a wide variety of deaths. 7	
6	Methods: these are clearly and concisely reported. The methods are appropriate to the objectives, though limited, as highlighted above.<br><br>Results: the results are presented clearly. It might be helpful to know the response rate: how many professionals from how many countries were emailed as members of ISPID or included in the ISPCAN CDR working group or as part of the Uppsala workshop?	We have clarified the number of countries invited to participate, it's not possible to calculate a response rate as we do not know how many people visited the ISPCAN CDR working group forum, or the exact number of those attending the ISCAN meeting. Many of those emailed via ISPID will not have had any involvement in CDR so appropriately did not respond to the survey.<br><br>We directly approached CDR professionals from 25 different countries via professional networks, there were 27 eligible responses provided, representing 19 different countries.	P4
7	I am not convinced that Box 1 adds anything to the results: why are just Styria (Austria) and Japan reported here? Boxes 2, 3 and 4 give a bit more detail, but it is unclear why just four of the 13 countries are included in each.	The boxes represent free-text comments relevant to the numerical data to illustrate the variation in maturity of CDR processes across several higher-income countries. Not all countries provided free text comments, hence only a few countries were included as examples. However we agree that Box 1 offers limited context from an international perspective so have removed this.	
8	Discussion: Generally, the discussion summarises the key findings, with some limited reflection on these. The authors make some important points about the value of reviewing all child deaths, having a multi-disciplinary approach, and of involving parents, along with the limitations of not having globally comparable data. There is some helpful consideration of the role of legal protection of professionals.<br><br>It is a bit misleading to state that the paper reports on CDR practices in 21 countries when for five of these the only data presented are that they are planning to develop CDR and facing difficulties in this.	Thankyou. We acknowledge that the original aim of the study could be tightened to reflect intention, and have clarified this to: the primary aim of this study is to compare key contemporary CDR practices to support development of international best practice guidelines achievable in different countries and regions within countries. However we believe that the description of the study as being a descriptive study designed to determine status, scope and operational processes of CDR practices in different countries, was inclusive of countries in the process of planning and implementing new CDR systems.	

9	In conclusion, the authors state that 'this study goes beyond documenting the current state of international CDR practices and shows the need for global standards that can ensure that each child death, while remaining a tragedy, are regarded as an opportunity for learning and prevention.' I am not convinced that it really does go beyond documenting the current state of international CDR practices, nor that it really achieves that given the limited involvement in the survey.	The concluding paragraph has been completely rewritten as follows:<br><br>In conclusion, this study highlights both the diversity of CDR practices internationally and the common principles that underpin them. Across settings, the shared purpose of CDR is to learn from child deaths and identify opportunities to strengthen systems, improve care, and prevent future deaths. How these principles are implemented should be shaped by local epidemiology, health priorities, available resources, and cultural context, rather than through a single standardized model. Our findings support the value of multidisciplinary review processes that promote learning from systematic factors in a culture of openness and continuous quality improvement, while recognizing that alternative mortality review approaches may be more appropriate in some settings. Future international collaboration, including broader representation from low- to middle-income countries and First Nations communities, could help define core principles for effective child death review while allowing flexibility in their implementation. Such work could inform future World Health Organisation guidance and strengthen the contribution of mortality review to child survival efforts globally.	P10
10	I am not convinced that 'Our findings indicate that child death reviews should function as a quality improvement tool'. Common sense and the defined purpose of CDR would suggest that, but your survey has not provided any evidence that this is a worthwhile aim or that CDR actually achieves it.	This has been rewritten as detailed in point 9	
11	The statement that 'Reducing child mortality rates worldwide requires robust CDR by multidisciplinary teams of professionals, transforming CDR data into a state strategy that directly informs legislative processes' also does not follow from your findings. Most reduction in child mortality worldwide has occurred prior to the establishment of CDR and through public health interventions that have emerged through different processes. To my knowledge, there is no evidence so far that CDR can or does reduce child mortality rates in any region.[	This has been rewritten to reflect the role of public health:<br><br>Public health interventions have contributed to a significant decline in global child mortality, potentially this decline could be further assisted by transforming data from robust multidisciplinary CDR into state strategies.	P10

12	The statement that 'we now need to update the World Health Organization CDR guidelines' may be true, but as you have not reported on or critiqued the current content of those guidelines, nor identified any specific features that need updating, this statement is not justified.	We have included earlier in the discussion where practice differs from the WHO guidelines, to justify the need for revision. We have rephrased our justification to tone it down.<br><br>Few countries involve parents in CDR which potentially limits learning. The World Health Organization CDR guidelines refer to 'sometimes' interviewing parents but do not clarify when this may or may not be appropriate. 8<br><br>Although all respondents reported multi-disciplinary participation in CDR, many had a very medical or forensic focus not including public health, social work or education. Similarly, the World Health Organization CDR guidelines only relate to review of deaths within paediatric healthcare facilities. 8 This lack of multi-disciplinary CDR suggests potential weakness in the analysis of social determinants of health; this is of concern as child mortality is strongly socially determined<br><br>Updated World Health Organization CDR guidelines, reflecting multidisciplinary CDR and the voices of bereaved parents could help transform CDR systems and help prevent future child deaths.	P10
	**Reviewer 2 (VP)**		
1	This is an important review of practice in child death review using empirical data from an international survey of programs. My major issues are (1) the authors need to expand any justification for why child death review should be more similar across jurisdictions with any variations being addressed (are they not adequately serving their constituents, justify reason for survey?),	This has been expanded on in the discussion:<br><br>Despite the same purpose for CDR there is a striking diversity of CDR in practice, similar to that noted previously. 1 22 This variation limits international collaboration and development of global standards, as there is no consensus on which deaths are reviewed, the format of reviews, or data collected. This is seen in the ISPCAN survey which asked for the percentage of child deaths due to different categories of abuse; this varies depending on the inclusion criteria for reviews with much lower levels in countries reviewing all deaths compared to those only reviewing deaths from abuse or neglect. 9 International CDR datasets collecting similar variables may have great value in advancing scientific knowledge of rare conditions such as unexplained infant and child deaths. There is considerable international	P8

		variation in the classification of sudden infant deaths, 23 with risk factors such as bed-sharing often interpreted as causal despite limited evidence hindering surveillance and research. 24 Common datasets would overcome these difficulties and potentially help with understanding rare poorly understood conditions such as sudden unexplained death in childhood (SUDC). Such datasets could also help elucidate the complex relationships between ethnicity, social deprivation and child mortality seen in many developed countries.	
2	and (2) formatting for the draft needs to be consistent in line with the journal's policies.	We have tried hard to format the paper to the journal's policies, we leave further formatting to the editors.	
3	Minor comments:<br><br>MC1. it would be helpful to add data form the International Society on the Prevention of Child Abuse and Neglect regarding countries' participating in CDR. Their publication World Perspectives has data on a good number of countries which can be summarized for the introduction;	This has been added to the introduction:<br><br>A survey conducted by ISPCAN (International Society for the Prevention of Child Abuse and Neglect) in 2019-2020 found that 27/62 countries had some form of child fatality review although there were no details on the systems or methods of review. 9	P3
4	MC2. The Figure 1 caption should be rephrased to include countries with no CDR;	Figure 1 is designed to only show countries that have CDR, so is captioned correctly.	
5	MC3 Box 2 should include some information about the US National CDR Center and how it amasses data across these different jurisdictions;	The original box 2 only relates to the legal basis for CDR. This information is already provided in the next box.	
6	MC4 It would be helpful to reorganize and number the issues: (1) Countries with programs, (2) Different aims for programmes, (3) Parental Involvement, (4)Legal claims/issues, (5) types of deaths reviewed; (6) outcomes of CDR, (7) process/ use of standard forms, etc.	We have provided sub-headings for these. We leave it for the editor to decide if these sections should be numbered.	
7	MC5 Expand and add separate section for limitations.	We have expanded the paragraph on limitations:<br><br>Despite the relatively small sample, our established international collaborations with CDR leaders enabled us to purposively recruit informants with detailed knowledge of CDR policy and practice within their countries or regions, providing confidence that the data accurately reflect the systems described. The international composition of the author team also brought diverse	P9

		disciplinary and jurisdictional perspectives to the interpretation of findings.	
8	MC6 The last paragraph has some good points that need to be brought out in the abstract and introduction. Include the WHO CDR guidelines in the Introduction.	The conclusion of the abstract has been rewritten:<br><br>Comon principles underpin effective child death review, providing a foundation for locally adapted approaches to improve systems and prevent future deaths.	
9	MC7 Add papers from the US National CDR Center (Collier et al., Pediatrics, 2024).	Some examples from the US National CDR Center have been included in the introduction:<br><br>High quality CDR should be a public health priority and has recently been endorsed as such by the American Academy of Pediatrics.7 The English National Child Mortality Database has conducted many different analyses of child deaths including those from suicide, 14 asthma15 and life-limiting conditions.16 The US National Fatality Review-Case Reporting System has supported several analyses for example deaths from suicide,17 drowning, 18 and opioid poisoning. 19 These show the potential value of detailed CDR and regional or national data collection systems.	P2-3
10	MC8 Use a figure for point 5 info on results in the appendix.	8. Use a figure for point 5 info on results in the appendix.<br><br>We don't think that a figure is needed in the appendix as the question and response options are clear. The results for point 5 are given in figure 2.	
11	MC9 Were the results of points 16, 18 in the appendix given in the paper?	The results of point 16: the professionals taking part in CDR and point 18: the use of standard templates is already reported in the results.<br><br>Professionals involved in CDR<br><br>A range of different professionals were involved in reviews, with paediatricians, social workers, forensic pathologists and public health personnel most commonly involved.<br><br>Format of CDR<br><br>Standard CDR forms or templates were used in 15 regions to assist reviews;	

		other regions were more flexible in their review processes	
12	MC10. Add total N in the figure 2 caption.	This has been added as suggested	
	REVIEWER 3 (SV)		
1	This is a clearly written paper that will add to the limited evidence base around international variations in child death review practice. I just have one query. The abstract and results section state that participants came from 19 different countries. However, the first line of the discussion section states that the survey reports on CDR practices in 21 countries so it's unclear where this figure comes from.	Thank you for noticing this. There were 20 responses from 13 different countries included. We have actually changed the first line of the discussion anyway.<br><br>This survey reports on the CDR practices in 13 countries	P8
