# Supplementary material for: Shared principles, local practice: an international survey of child death review systems
Source: BMJ Paediatr Open. 2026 Sep 8;10(1):e005019. doi: 10.1136/bmjpo-2026-005019 (PMC13561003; doi:10.1136/bmjpo-2026-005019)
Supplement: Supplementary file 2 [file bmjpo-10-1-s001.docx]

**Appendix 1 International CDR survey questions**

1. Which country do you work in?
2. Child Death Review (CDR) is the multi-professional review of child deaths aiming to identify causes, risk factors and learning from deaths; with the aim of improving services and preventing future deaths. Is there Child Death Review for any type of child death in your country? Yes/No
3. Do CDR practices vary by region in your country? Yes/No
4. Which region, state or county do you work in? Please answer all the following questions based on the CDR practices in your region, state or county, where possible
5. What are the aims of CDR in your region? Tick all that apply
   a. To identify individual children’s cause of death
   b. To identify risk factors for death
   c. To identify learning to prevent future deaths
   d. To inform public health campaigns
   e. To improve healthcare and other services
   f. To provide information and support for bereaved families
   g. To code deaths in a standardised manner for regional or national analysis
   h. To provide data for research projects
   i. Other – please state
6. Is CDR mandated by national or regional laws or required by local policy? Yes/No
7. Is there national collection of data from CDR? Yes/No
8. In the region where you work, are parents informed that there will be a review of their child’s death?
   a. Yes - parents are always or nearly always told about CDR
   b. Sometimes parents are told about CDR
   c. No - parents are never or almost never told about CDR
   d. I don't know if parents are told about CDR
9. In the region where you work is parental consent to share information for CDR required? Yes/ No/ Other - please state
10. In the region where you work, in addition to any reports or statements for Coroners or police investigations, are parents invited to ask questions or give feedback for the CDR meeting? Yes/No
11. In the region where you work can parents provide information or comments for the CDR? Yes/No
12. In the region where you work are the findings from CDR shared with parents? Yes/ Sometimes/ No/ Other - please state
13. In the region where you work are professionals participating in CDR protected from malpractice or other legal claims? Yes/No
14. Which types of child death are reviewed in the region where you work? Tick all that apply. In each category: Yes ALL / Yes SELECTED/ None
    a. All child deaths
    b. Sudden unexpected or unexplained or infant deaths - under 12 months of age (SUID/SUDI)
    c. Sudden unexpected or unexplained child deaths - over 12 months of age (SUDC/SUDIC)
    d. Deaths from child abuse or neglect
    e. Death from medical conditions occurring in hospital
    f. Death from medical conditions at home with palliative care or in a hospice
    g. Suicide or self-harm
    h. Neonatal deaths
    i. Stillbirth
    j. Homicide
    k. Deaths from trauma
    l. Other – please state
15. Which age of children are included in CDR in your region? Tick all that apply
    a. Under 1 year
    b. 1-5 years
    c. 6-10 years
    d. 11-17 years
    e. Other – please state
16. Which professionals take part in CDR? Tick all that apply
    a. Paediatricians
    b. Pathologists
    c. Police
    d. Social workers
    e. Nurses
    f. Public health
    g. Education
    h. Coroner
    i. Medical Examiner
    j. Other – please state
17. In your region, how long after the death does the CDR usually take place?
    a. Less than 6 months
    b. 6- 12 months
    c. 1-2 years
    d. More than 2 years
18. In your region, do you use a standard template or form for completing reviews? Yes/No/Sometimes/Other please state
19. Do you have any further comments on CDR in your region or country?
